# Removal Efficiency of Radioactive Cesium and Iodine Ions by a Flow-Type Apparatus Designed for Electrochemically Reduced Water Production

**DOI:** 10.1371/journal.pone.0102218

**Published:** 2014-07-16

**Authors:** Takeki Hamasaki, Noboru Nakamichi, Kiichiro Teruya, Sanetaka Shirahata

**Affiliations:** Department of Bioscience and Biotechnology, Faculty of Agriculture, Kyushu University, Higashi-ku, Fukuoka, Japan; Dowling College, United States of America

## Abstract

The Fukushima Daiichi Nuclear Power Plant accident on March 11, 2011 attracted people’s attention, with anxiety over possible radiation hazards. Immediate and long-term concerns are around protection from external and internal exposure by the liberated radionuclides. In particular, residents living in the affected regions are most concerned about ingesting contaminated foodstuffs, including drinking water. Efficient removal of radionuclides from rainwater and drinking water has been reported using several pot-type filtration devices. A currently used flow-type test apparatus is expected to simultaneously provide radionuclide elimination prior to ingestion and protection from internal exposure by accidental ingestion of radionuclides through the use of a micro-carbon carboxymethyl cartridge unit and an electrochemically reduced water production unit, respectively. However, the removability of radionuclides from contaminated tap water has not been tested to date. Thus, the current research was undertaken to assess the capability of the apparatus to remove radionuclides from artificially contaminated tap water. The results presented here demonstrate that the apparatus can reduce radioactivity levels to below the detection limit in applied tap water containing either 300 Bq/kg of ^137^Cs or 150 Bq/kg of ^125^I. The apparatus had a removal efficiency of over 90% for all concentration ranges of radio–cesium and –iodine tested. The results showing efficient radionuclide removability, together with previous studies on molecular hydrogen and platinum nanoparticles as reactive oxygen species scavengers, strongly suggest that the test apparatus has the potential to offer maximum safety against radionuclide-contaminated foodstuffs, including drinking water.

## Introduction

The Great East Japan Earthquake of magnitude 9 struck the northeastern coast of Japan on March 11, 2011. The earthquake caused a catastrophic tsunami, with the wave height of nearly 40.5 m, which caused failures in the nuclear reactor cooling system in the Fukushima Daiichi Nuclear Power Plant (FDNPP) [1], [2]. Soon after, these failures triggered hydrogen explosions in the nuclear reactors, discharging radioactive steam and liberating various radionuclides into the air over several days [2], [3]. Following the incident, natural factors such as wind flow, air streams, and rainfall caused dispersion and precipitation of various levels of radionuclides on land surfaces and vegetation in the Tohoku and Kanto regions [4]–[8]. Radionuclides were also detected in Fukuoka, 1,000 km away from the FDNPP [9], indicating the wide spread of the radioactive plume over Japan. Urgent action to cope with the situation involves decontamination of terrestrial and aquatic radioactivity sources, including drinking water. Incineration of contaminated materials such as plants, wood bark, garbage, and house wreckage is one choice for disposition, although it leaves cesium-enriched ash. An entire system for safe incineration, removal of ash radioactivity and safe disposal has been reported, with promising results [10]. Numerous conventional methods using ion exchange, various membrane processes, coagulation and co-precipitation and other technologies for eliminating radionuclides from radioactive wastewaters have been reported to be effective [11], [12]. Numerous approaches have been shown to remove radionuclides from contaminated water, including a mixture of activated carbon and/or zeolite-based media [13]–[15], co-precipitation with zinc hexacyanoferrate (II) followed by precipitation [16], sorption of radionuclides with biomaterials such as diatomite [17], Prussian blue immobilized diatomite or alginate/calcium beads or magnetic nanoparticles [18]–[20], arca shell [21], sulphuric acid-modified persimmon waste [22], nickel (II) hexacyanoferrate (III) functionalized walnut shell [23], mesoporous silica monoliths conjugated with dibenzo-18-crown-6 ether [24], and cobalt ferrocyanide impregnated anion exchange beads [25]. Additionally, a layered chalcogenide with a CdI_2_ crystal structure for adsorbing several cations has been explored [26].

Although these technologies are encouraging for removal of various levels of radionuclides and further improvements are expected to arise in the future, securing safe drinking water is also of prime importance. Rainwater samples collected in Fukushima in early April, 2011 have been reported to contain ^131^I (1470±26.5 Bq/L), ^134^Cs (100±25.3 Bq/L) and ^137^Cs (129±9.47 Bq/L) [27]. The fallout contaminates surface waters, including lakes and rivers, which are the main sources for preparing tap water to supply the residents in these regions. As a result, drinking water prepared from several water purification plants was reported to be contaminated. Subsidiary methods to reinforce conventional water purification systems have been reported to eliminate radioactivity from contaminated water sources. The efficacies of the coagulation-flocculation-sedimentation method in water purification plants, with removal efficiencies of 17% and 56% for ^131^I and ^134^Cs, respectively [28], [29], and radionuclide absorption by algal strains for environmental remediation [30], [31] have been assessed. Another significant point to consider is the contamination of drinking water via distribution system such as pipes, storage tanks, water pumps and heaters, which may be persistent contaminating sources. A recent review concluded that cesium appears to be removed by flushing water pipes with a low pH solution containing sodium or magnesium as ion competitors [32]. However, further assessment will be required before applying this approach to the vast areas of regional contamination. Approximately one month later, the radioactivity levels had decreased to below the limit values in the water purification plants [33]. Whereas even after 2 years, total Cs radioactivities above the limit values are reported in some foodstuffs, such as Chinese mushrooms, rice, soybean, adzuki-bean and several fish obtained from the areas surrounding the FDNPP [34], [35]. Moreover, low levels of radioactive Cs species are still detected in the drinking water of many cities around FDNPP [36]. These results imply that the fallout still remains on land surfaces and nearby mountain areas and that rainfall wash down is a highly probable contaminant of tap water sources [7], [8]. Precautions to avoid consumption of such foodstuffs, including drinking water, have been taken by measuring radioactivity levels prior to distribution. Nevertheless, following the accident, the concentrations of ^131^I in the tap water distributed by these purification plants were 210 Bq/L in Tokyo, 189 Bq/L in Ibaraki, and 220 Bq/L in Chiba, all of which exceeded the upper limit of ^131^I concentration set as 100 Bq/L for infants under 1 year of age by the Ministry of Health, Labour and Welfare, 1947 [3], [37]. Therefore, it is highly desirable to have terminal security systems that can achieve the removal of even lower levels of radioactive contaminants in tap water because, for example, radiocesium accumulates in the body. However, only limited studies examining removability of radionuclides from household water purifiers are available to date. Several domestic pot-type water purifiers have been suggested as a possible final security treatment to eliminate contaminated radionuclides in tap water [27], [38]. Although most of these pot-type water purifiers are efficacious, with varying degrees of radionuclide removal from contaminated water, they are useless against the biological effects exerted by unconscious ingestion of radionuclides via drinking water and/or foodstuffs.

Ionizing radiation emitted by ingested radionuclides causes water radiolysis by acting on the water molecules, which comprise approximately 80% of body weight [39]. Water radiolysis yields a variety of reactive oxygen species (ROS) including hydrogen peroxide (H_2_O_2_), the hydroxyl radical (^•^OH), superoxide anion radicals (^•^O_2_^−^), and other molecular species [40]. These free radicals cause extensive oxidative damage to biologically critical macromolecules such as DNA, RNA, proteins and lipids [41]–[45]. Such damage eventually induces cellular apoptosis or carcinogenic transformation [46], [47]. Therefore, an ideal apparatus should have the potential to provide both the elimination of radionuclides prior to ingestion and protection from detrimental ROS effects generated by the accidentally and/or unconsciously internalized radionuclides.

Considering these requirements, an apparatus designed to produce electrochemically reduced water (ERW) could be thought to fulfill such demands because it contains two functional units; an electrolysis unit for molecular hydrogen enrichment, and a micro-carbon carboxymethyl (CM) cartridge unit for removing various impurities. ERW produced from tap water by this apparatus contains as much as 0.587 ppm dissolved hydrogen (Table 1, [48]). Dissolved molecular hydrogen has been shown to exert a radioprotective effect in both *in vitro* and *in vivo* studies [49], [53]. These compelling results strongly support the suggestion that molecular hydrogen dissolved in ERW could function as a radioprotective agent in the body. Moreover, ERW was shown to contain platinum nanoparticles (Pt NPs) at up to 2.5 ppb as an ROS scavenger, liberated from Pt-electrodes during electrolysis [39], [54].

**Table 1:** Characteristics of the sample waters.

			ERW			
	TapWater	FilteredWater	Lv 1	Lv 2	Lv 3	Lv 4
pH	7.6±0.0	7.6±0.0	8.0±0.0	8.5±0.0	9.1±0.0	9.4±0.1
ORP (mV)	555.3±15.5	550.0±20.1	140.0±5.0	110.0±7.5	−673.3±2.5	−688.0±9.5
EC (ms/m)	49.3±0.1	49.5±0.1	49.7±0.1	49.7±0.1	49.0±0.2	48.1±0.2
DH (ppb, µg/l)	N.D.	N.D.	70.0±19.3	163.3±18.0	321.7±47.5	587.0±44.6
DO (ppm, g/l)	7.5±0.0	7.5±0.0	7.5±0.1	7.1±0.1	6.6±0.2	6.1±0.3

Filtered water: tap water was passed through the micro carbon cartridge without electrolysis. Lv 1: electrochemically reduced water (ERW) generated by electrolyzing the filtered water at level 1 with constant electric current at 50 volts (V) upper limit voltage and a flow rate of 1.8–2.0 l/min. Likewise, other ERWs were produced using identical conditions, except selecting the Lv 2 to Lv 4 switch. ORP: oxidation-reduction potential. EC: electrical conductivity. DH: dissolved hydrogen. DO: dissolved oxygen. Measurements were conducted at ambient temperatures. N.D.: Not Detected.

As for the second requirement, a micro-carbon CM cartridge unit composed of a nonwoven-fabric filter, several types of activated carbon and an ion-exchange material was present in the current test apparatus to remove particulate matters, microorganisms and 13 designated impurities [55]. However, this micro-carbon CM cartridge has not been assessed for its ability to remove radionuclides from contaminated tap water. Therefore, the present research was aimed at evaluating whether the test apparatus as a whole is capable of removing radionuclides from contaminated tap water.

## Materials and Methods

### Chemicals

Cesium chloride (CsCl) and potassium iodide (KI) were purchased from Wako Pure Chemical Industries (Osaka, Japan).

### Radioisotopes

^137^CsCl [0.2021 MBq/g] and Na^125^ I [12.950 TBq/g] were purchased from Japan Radioisotope Association (JRIA, Tokyo, Japan). We used ^125^I because Kyushu University Radioisotope Center has an approval to use this radionuclide. Tap water distributed by the Fukuoka City Waterworks Bureau, Fukuoka, Japan was used in all experiments except ultrapure water (Milli Q water, Merck Millipore, Tokyo, Japan) for the preparation of standard solutions for inductively coupled plasma-mass spectrometry (ICP-MS) analysis.

### Electrochemically reduced water (ERW)-producing apparatus

**Figure 1:**
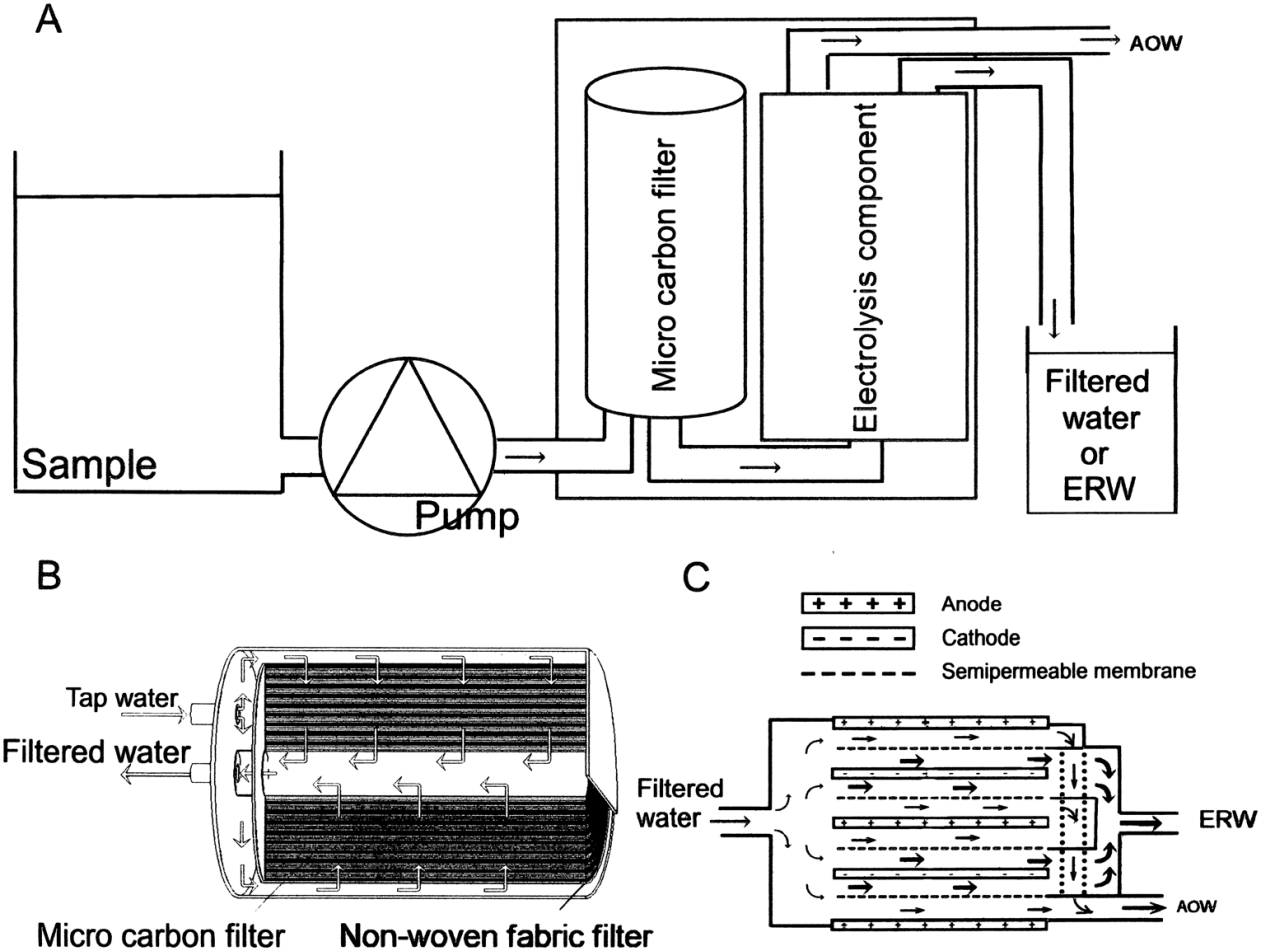
Schematic of the flow-type electrolysis apparatus. The test apparatus is composed of two units, a micro-carbon CM cartridge (B) and an electrolysis unit (C). The overall water flow and equipment set up is shown in (A). Sample water is connected to an adjustable speed pump to maintain a flow rate of 1.8–2.0 l/min and expelled to the inlet of the electrolysis unit (A). Tap water passes through the nonwoven-fabric filter, the mixed layers of activated charcoal powders and cationic ion-exchange material to make filtered water (B). Filtered water then flows into the electrolysis unit composed of platinum-coated 5 electrode plates separated by semi-permeable membranes (C). Filtered water will be electrolyzed at levels 1, 2, 3 and 4 at a maximum of 50 volts while passing through the gaps between the electrodes.

A water flow-type apparatus, Trim Ion NEO, was provided by Nihon Trim Co. Ltd., Osaka, Japan as the test apparatus. This test apparatus is composed of two units, a micro-carbon CM cartridge unit (Fig. 1B) and an electrolysis unit (Fig. 1C). Tap water flows into the cartridge unit, where tap water passes through the nonwoven-fabric filter to remove macroparticles, and pre-cleaned water flows into mixed layers of activated charcoal powders and cationic ion-exchange material to remove most of the impurities, including dissolved lead and 13 other elements that must be removed. The remaining contaminants, such as microorganisms and iron rust particles larger than 0.1 µm in size, are also eliminated by the cartridge (Fig. 1B). The micro-carbon CM cartridge unit is certified to withstand filtration of at least 12 tons of tap water per year or 35 liters per day for 1 year. In the present study, we used a new cartridge unit for each experiment. Purified tap water flows into the electrolysis unit, which is composed of five platinum (Pt)-coated electrode plates, separated by semi-permeable membranes and the water is electrolyzed while passing through the gaps between the electrodes (Fig. 1C). Platinum-coated titanium electrodes are certified for at least 1,400 hours use without a marked deterioration with respect to the efficacy of water electrolysis, suggesting that the loss of a small amount of Pt nanoparticles from the surface of the electrode will not significantly affect the electrolysis efficacy of the device used here. Electrolyzed tap water near the cathode typically exhibits a high pH, low dissolved oxygen, high negative redox potential and a high concentration of dissolved hydrogen (0.4–0.9 ppm) (Table 1, [48]). Water produced in this manner, with the above characteristics, is designated as ERW. The test apparatus is designed to produce five types of water; four types of ERW (Levels 1–4) electrolyzed with a constant electric current for each level (0.8 to 4.2 A) at a maximum of 50 volts and one type of filtered water without electrolysis (Table 1). ERW is produced near the cathode, as indicated by the thick right-facing arrows in Fig. 1c, and positively charged radioactive Cs ions will be attracted to the cathode side during electrolysis, resulting in an increased concentration of Cs^+^ ions in ERW, dependent upon the current intensity. Conversely, negatively charged I ions will be attracted to the anode side, resulting in a decreased concentration of I ions in ERW. The electrolysis currents were increased in the order of levels 1 to 4, where Level 4 represents the strongest current, reflecting the highest dissolved hydrogen (DH) and the lowest oxidation-reduction potential (ORP) (Table 1). When the radioactivity of ERW at level 4 is measured as being lower than the background level, then one can conclude that the radioactivity of ERW at levels 1 to 3 is lower than the background level. ERW at levels 1 to 3 is usually used for drinking and at level 4 is used for cooking. We have included Table 1 to aid the readers understanding of the four types of ERW.

### Preparation of non-radioactive sample water (CsCl, KI)

Tap water was used as a control. CsCl solutions of 20 liters each with concentrations of 20 and 2,000 ppb were prepared using tap water. Likewise, KI solutions with concentrations of 100 and 4,000 ppb were prepared. These solutions are designated as sample waters. The test system was arranged by placing an adjustable speed pump between the sample waters and the test apparatus to mimic tap water pressure, connected to the inlet of the test apparatus, as shown in Fig. 1A. The water flow rate was set to 1.8–2.0 L/min by adjusting the pump speed throughout the entire experiment. In the experiment, 1–2 liters of tap water was used to wash and equilibrate the system each time the sample concentrations were changed. Fifteen milliliters of filtered, ERW and relevant control waters were collected for ICP-MS analysis. The removal efficiency was calculated according to a previously described equation [38], shown in Tables 2 and 3.

**Table 2:** Removal efficiencies (%) for Cs ion and ^137^Cs.

Measured (Loaded) amounts		Removal efficiency (%)
as Cs ion (ppb)	as ^137^Cs (Bq/kg)	
1976.47 (2000)	0	58.2
20.55 (20)	0	87.4
^#^3.1600	16212.0 (15000)	96.9
*^#^*0.6360	3262.0 (3000)	96.9
^#^0.0642	329.0 (300)	99.2*
^#^0.0067	34.9 (30)	92.5*

Removal efficiency (%) = (1−[A]/[B])×100 according to [38]. [A], [B]: concentrations of Cs and ^137^Cs after and before filtration. Each solution was filtered only, without electrolysis. *: [A] values were below the detection limit. *: [A] values used to calculate removal efficiency were below the detection limit. ^#^: equivalent ppb values calculated from the radioactivities loaded. Values within parentheses were prepared and loaded amounts or radioactivities of cesium.

**Table 3:** Removal efficiencies (%) for I and ^125^I ions.

Measured (Loaded) amounts		Removal efficiency (%)
as I ion (ppb)	as ^125^I (Bq/kg)	
3891.0 (4000)	0	84.6
130.0 (100)	0	91.7
^#^0.0000197	14993.0 (15000)	99.4
^#^0.00000351	1788.0 (1500)	99.3
^#^0.000000196	146.3 (150)	99.5*

Removal efficiency (%) = (1−[A]/[B])×100 according to [38]. [A], [B]: concentrations of I and ^125^I solutions after and before filtration. Each solution was filtered only, without electrolysis. *: [A] values used to calculate removal efficiency were below the detection limit. ^#^: equivalent ppb values calculated from the radioactivities loaded. Values within parentheses were prepared and loaded amounts or radioactivities of iodine.

### ICP-MS analysis of Cs and I elements in ERWs

Sample waters were passed through the apparatus, and collected filtered waters were quantitated using ICP-MS (Agilent 7500c, Agilent Technologies Co. Ltd., Santa Clara, CA, USA) in the Radioisotope Center at Kyushu University.

### Preparation of radioactive sample water (^137^CsCl and Na^125^I)

Stock solution of ^137^CsCl was diluted with 20 liters of tap water to prepare concentrations of 15,000, 3,000, 300, and 30 Bq/Kg. Likewise, Na^125^I stock solution was diluted with 20 liters of tap water to prepare concentrations of 15,000, 1,500, and 150 Bq/Kg. All other experimental conditions, such as water flow rate, system equilibration, the electrolysis conditions of the apparatus were carried out as closely as possible to those used for the non-radioisotope experiments, except that 10 ml of each of the sample waters were collected for radioactivity counting.

### Radioactivity counting of ^137^Cs and ^125^I in sample waters

Radioactive sample waters were passed through the apparatus, and collected waters were quantitated using a gamma counter (AccuFLEX γ ARC-7001, Hitachi Aloka Medical, Ltd., Tokyo, Japan) in the Center of Advanced Instrumental Analysis at Kyushu University. To evaluate the effect of the electrolysis step on radionuclide removal, filtered waters were electrolyzed by a constant current (4.2 A) at level 4 and radioactivities of ERW were quantitated as above.

### Statistical analysis

All experiments were performed in triplicate. Data are expressed as means ± SD for each experiment.

## Results

### Analysis of Cs and I elements in the filtered water

**Figure 2:**
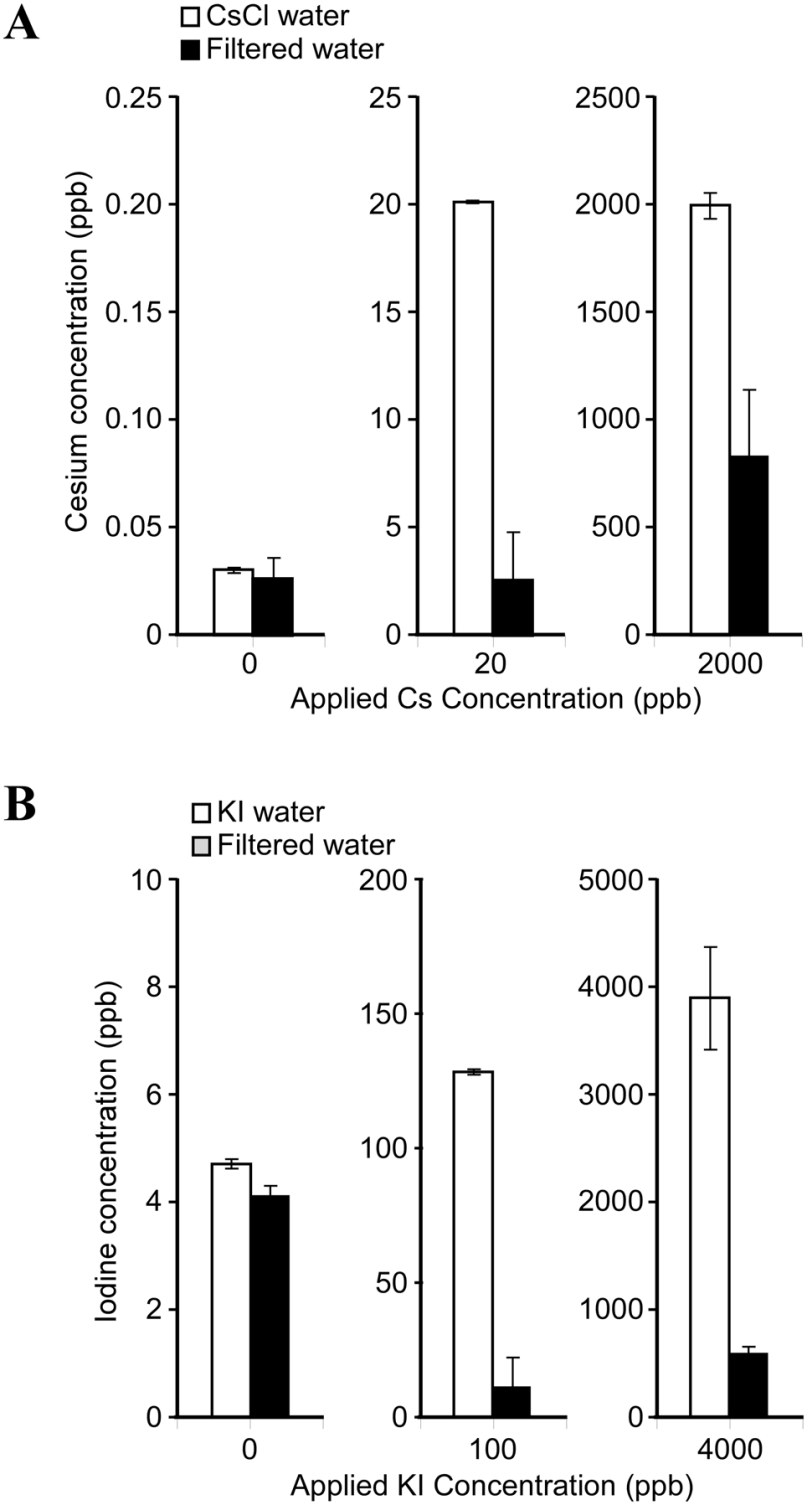
Measurement of Cs and I elements in filtered waters. CsCl solutions at concentrations of 0, 20 and 2,000 ppb were passed through the test apparatus. Collected filtered waters were used to measure Cs concentration by ICP-MS (A). KI solutions at concentrations 0, 100 and 4,000 ppb were passed through the test apparatus. Collected filtered waters as in (A) were used to measure I concentration by ICP-MS (B). White bar: Tap water, gray bar: Filtered water. Experiments were carried out in triplicate.

Prior to radioisotope experiments, CsCl and KI solutions were prepared as described in the Materials and Methods section and their removability was tested. The background Cs concentration in tap water was similar to that for the filtered water (Fig. 2A, column 0 ppb). When 20 and 2,000 ppb CsCl solutions were used, the measured values of the filtered water indicate that the test apparatus had a higher removability (87.4%) for the 20 ppb CsCl solution than for the 2,000 ppb CsCl solution (58.2%) (Fig. 2A, Table 2). Similar experiments using KI solutions were carried out and the results are shown in Fig. 2B. The background I concentrations in tap water and that for the filtered water were similar (Fig. 2B, column 0 ppb). Removal efficiency after filtration for 100 ppb and 4,000 ppb KI solutions were 91.7% and 84.6%, respectively (Table 3). These results demonstrate that the micro-carbon CM cartridge is capable of removing Cs and I ions at all concentration ranges tested (Fig. 2).

### Removal efficiency of ^137^CsCl and Na^125^I in the filtered water

**Figure 3:**
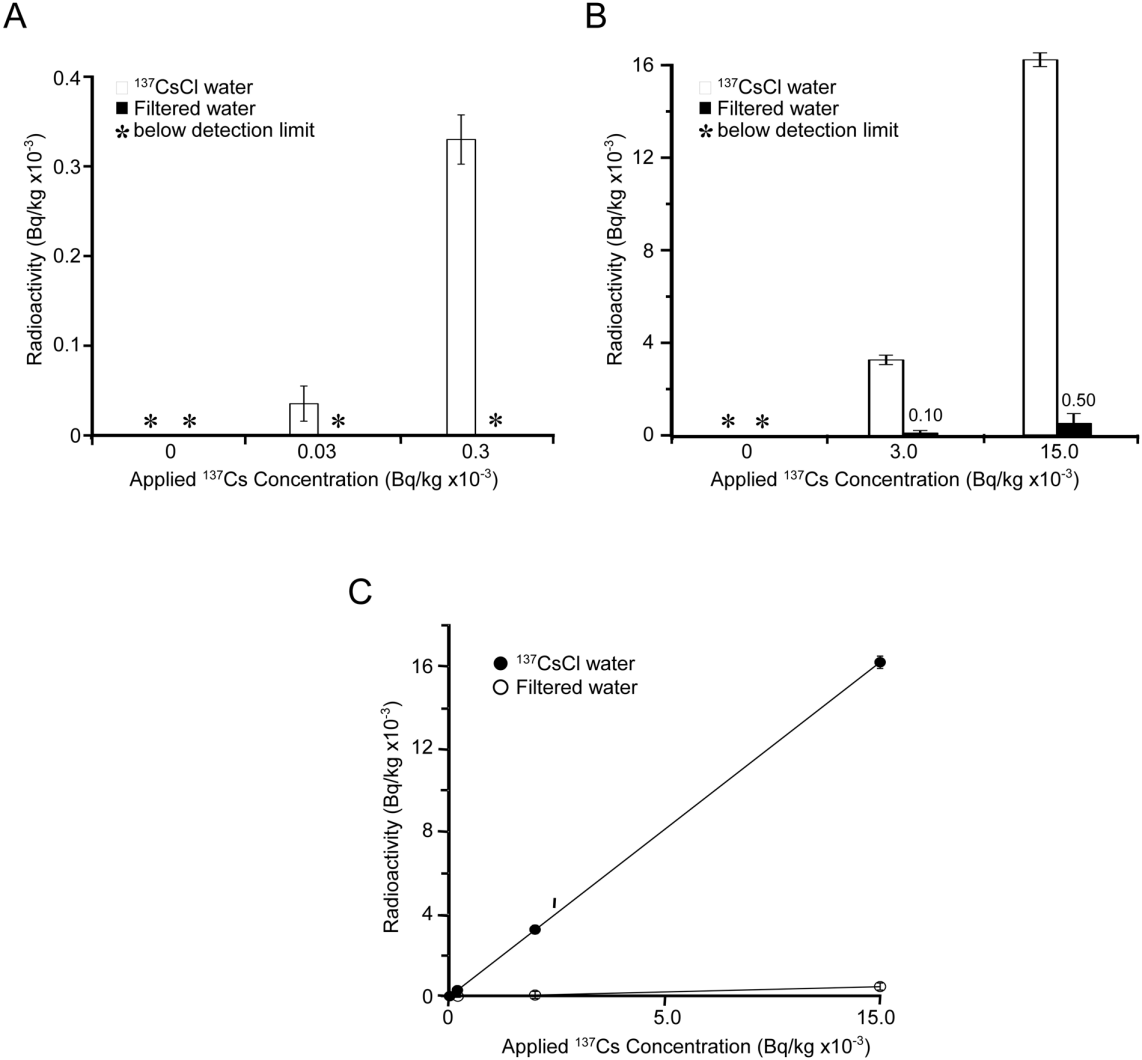
Measurement of ^137^Cs in sample waters. ^137^CsCl solutions at concentrations of 0, 0.03, 0.3, 3.0 and 15.0 KBq/kg were passed through the test apparatus. Collected filtered waters were used to measure ^137^Cs counts by an AccuFLEX γ ARC-7001 gamma counter (A and B). White bar: ^137^CsCl solutions before filtration, gray bar: ^137^CsCl solutions after filtration. Radioactivities before and after filtration were evaluated by linear-regression analysis (C). •: ^137^CsCl solutions before filtration, ○: ^137^CsCl solutions after filtration. Experiments were carried out in triplicate.

Because the test apparatus removed Cs and I ions efficiently, assays were extended to examine the removability of ^137^CsCl and Na^125^I. The natural background counts in tap water and filtered water were below the detection limit of the gamma counter (Fig. 3A and B, column 0). Tap water containing 30 (0.0067 ppb as Cs ions), 300 (0.0642 ppb as Cs ions), 3,000 (0.636 ppb as Cs ions) and 15,000 (3.16 ppb as Cs ions) Bq/kg of ^137^CsCl as controls showed the expected radioactive counts (Fig. 3A and 3B, white bar at each concentration) with a high correlation coefficient (Fig. 3C, R ^2^ = 0.999). Control waters were then passed through the micro-carbon CM cartridge and the filtrate radioactivities were measured (Fig. 3A and B). It was found that the radioactivities of the filtered water for ^137^CsCl were reduced significantly (Fig. 3A and 3B) and removal efficiency was 96.9%, even after loading 15,000 Bq/kg of ^137^CsCl (Table 2).

**Figure 4:**
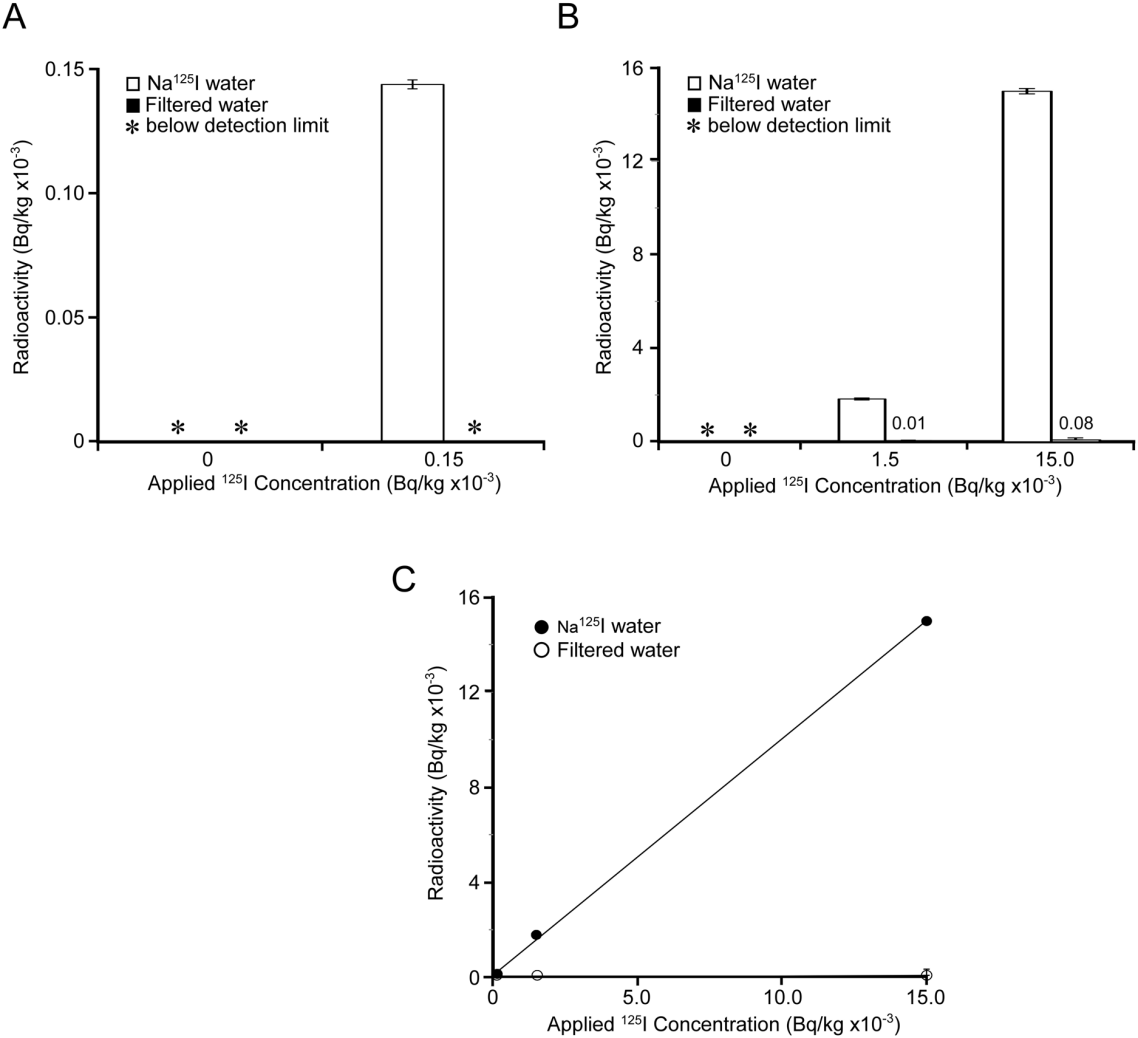
Measurement of ^125^I elements in sample waters. Na^125^I solutions at concentrations of 0, 0.15, 1.5 and 15.0 KBq/kg were passed through the test apparatus. Collected filtered waters were used to measure ^125^I counts by an AccuFLEX γ ARC-7001 gamma counter (A and B). White bar: Na^125^I solutions before filtration, gray bar: Na^125^I solutions after filtration. Radioactivities before and after filtration were evaluated by linear-regression analysis (C). •: Na^125^I solutions before filtration, ○: Na^125^I solutions after filtration. Experiments were carried out in triplicate.

To evaluate Na^125^I removability, we prepared Na^125^I containing sample waters as described above. The natural background count in tap water and filtered water exhibited values below the detection limit (Fig. 4A and B, column 0). Tap water containing 150 (0.000196 ppt as I ions), 1,500 (0.00351 ppt as I ions) and 15,000 (0.0197 ppt as I ions) Bq/kg of Na^125^I as controls showed expected radioactive counts (Fig. 4A and 4B, white bar at each concentration) with a high correlation coefficient (Fig. 4C, R ^2^ = 0.999). Radioactive control tap waters were passed through the micro-carbon CM cartridge, reducing the filtrate radioactivities significantly (Fig. 4A and B), with a removal efficiency of over 99% (Table 3). Thus, the micro-carbon CM cartridge was demonstrated to efficiently remove radioactivities up to 15,000 Bq/kg of Na^125^I.

### Effect of electrolysis on the removal efficiency of ^137^Cs and ^125^I

**Figure 5:**
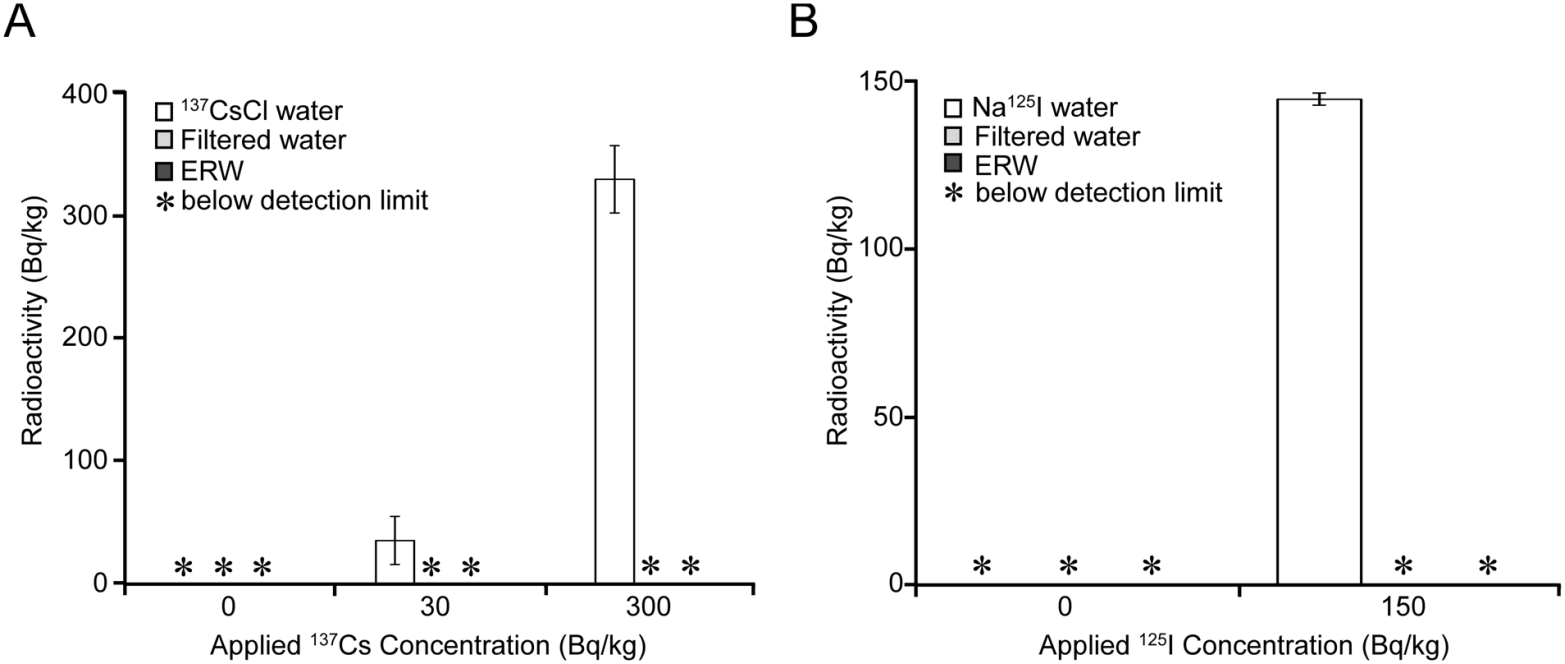
Effects of electrolysis on filtered radioactive sample waters. ^137^CsCl solutions of 30 and 300 Bq/kg were passed through the test apparatus and filtered waters were collected for measurement. Then, filtered water was passed through the electrolysis unit at the highest electrolysis level of 4 and ERW was collected for measurement. Collected waters were used to measure ^137^Cs counts by an AccuFLEX γ ARC-7001 gamma counter (A and B). Using the same protocol, filtered water and ERW were collected for 150 Bq/kg of Na^125^I solution. White bar: ^137^CsCl or Na^125^I solutions, gray bar: Filtered ^137^CsCl or Na^125^I solutions; black bar: ERWs of filtered ^137^CsCl or Na^125^I solutions. Experiments were carried out in triplicate.

In parallel with the preceding experiments, we evaluated the effects of the electrolysis step in terms of efficiencies for ^137^Cs and ^125^I removal from the filtered radioactive water. Filtered water was electrolyzed at the highest current level of 4. In this experiment, we selected 300 Bq/kg of ^137^Cs water, which loaded 30 times more radioactivity than the upper limit value of 10 Bq/kg for drinking water set by the government [56]. Under these conditions, the radioactivity in ERW remained below the detection limit (Fig. 5A). Similarly, we evaluated the removability of ^125^I by the highest electrolysis level of 4. In this case, we selected 150 Bq/kg of ^125^I, which is a loading of 1.5 times more radioactivity than the upper limit of 100 Bq/L of ^131^I concentration for infants under 1 year of age set by the Ministry of Health, Labour and Welfare, 1947 [37]. The radioactive iodine level in ERW remained below the detection limit (Fig. 5B). Therefore, the results indicate that the cartridge substantially removed ^137^Cs and ^125^I from tap water prior to the electrolysis step, thereby assuring undetectable levels of radioactivity in ERW produced at the highest current level of 4, which has the highest attraction for ^137^Cs^+^, and thus the results hold true for ERWs produced with the current levels 1 to 3.

## Discussion

The FDNPP accident liberated various radionuclides, including ^131^I, ^132^I, ^134^Cs, and ^137^Cs [57]. Amongst these radionuclides, ^131^I can enter the body through inhalation and by ingesting contaminated foodstuffs including drinking water, which then rapidly concentrates in the thyroid gland, where β-radiation exposure takes place. As its half-life is 8 days, radioactivity levels are expected to be reduced substantially over several months. Therefore, an obvious precaution is not to ingest ^131^I-contaminated or doubtful foodstuffs including drinking water. Water supply law in Japan limits the lowest chlorine concentration in tap water outlet at 0.1 mg/L [58]. Dissolved ^131^I is reported to form various species in tap water such as the radioactive iodide ion (^131^I^−^), hypoiodous acid (HO^131^I), the iodate ion (^131^IO_3_^−^), iodine molecules (^131^IO_2_) and organic ^131^I. ^131^I^−^ reacts with chlorine and is transformed mainly into HOI at neutral pH. HOI is further transformed into IO_3_^−^ by reacting with chlorine [29], and as a result, almost all iodine is converted to the iodate ion (IO_3_^−^) in tap water due to the oxidation by chlorine [59]. It is reported that ^131^I^−^ removal is increased by water containing 0.1–0.5 mg/L chlorine, with lower concentrations of powdered activated charcoal [29]. However, granular and powdered activated carbons were reported to remove ^131^I at about 30–40% efficiency. Additionally, it has been reported that ^125^I^−^ and ^125^I_3_^−^ were prepared from ^125^I and used to test the removability of these species by a granular type charcoal, which resulted in a small amount of adsorption [60]. These results may partly explain the inefficient removability by activated charcoal reported by others, through selective adsorption of iodate and iodine [38], [60], [61]. Activated carbon was shown to remove iodide (I^−^) more efficiently than iodate (IO_3_^−^) [27]. Therefore, it appears that combinations between the types of activated carbon/charcoal and iodine species affect overall removability. In the present experiments, we used tap water distributed by the Waterworks Bureau of the City of Fukuoka, expected to contain at least 0.1 mg/L chlorine. Thus, ^125^I is mostly, if not completely, converted to iodate ions (IO_3_^−^) by chlorine in the tap water. In the present results, KI and ^125^I were efficiently removed from tap waters by the micro-carbon CM cartridge, suggesting that iodide and iodate ions were removed. The micro-carbon CM cartridge is composed of a nonwoven-fabric filter and activated carbons consisting of a coconut shell activated carbon powder, a coconut shell activated carbon conjugated with a silver compound for antimicrobial effect, and an amorphous titanosilicate-based inorganic compound (BASF Co, Germany) molded with a fibrous binder for shaping. This cartridge was used in the present test apparatus to remove particulate matters, microorganisms, and for qualified removability of 13 designated impurities, tested according to the standard method set by JIS S 3201, 2004 (Domestic Water Purifier Quality Test) [55]. It is worth mentioning that the test apparatus effectively removed I and ^125^I (applicable to Cs^+^ and ^137^Cs), even though water was supplied to the apparatus through a pump simulating tap water outlet pressure to attain 1.8–2.0 L/min flow rate, which markedly reduced the contact time of water with the activated carbon surfaces and ion-exchangers compared with those in pot-type water purifiers. It has been reported that the above-mentioned molded activated carbons can replace the hollow fiber membrane filter that is commonly used in other water purifiers to eliminate materials larger than 0.1 µm in size [55]. Incidentally, hollow fiber membranes do not contribute to the elimination of iodate (IO_3_^−^) ions because their radius is 0.326 nm, even when their radius is increased several fold in water [61]. Additionally, the ineffectiveness of removing ^131^I by boiling tap water has been reported [3].

Cesium is an alkaline earth metal that exists as a monovalent cation form (Cs^+^) in water and in soils [27]. We found that Cs^+^ could be efficiently removed by the micro-carbon CM cartridge tested here. The mechanism for the removal of Cs^+^ remains to be investigated. The Cs^+^ removal efficiency by the apparatus was 87.4% at 20 ppb, which is comparable to that of several pot-type water purifiers that have efficiencies of around 90% for tap water containing 40–50 µg/l (ppb) cesium chloride [38]. A removal efficiency of 58.2% for Cs^+^ appears to be low at the highest concentration (1976.5 ppb) loading. This lower removal efficiency could be explained by the amount of Cs^+^ getting close to system over loading because this amount is 625.3 times more Cs^+^ ion loading than the 3.16 ppb Cs^+^ ion calculated from the highest radioactivity (16,212 Bq/kg) loading where 96.9% removability was attained (Table 2). Therefore, the apparatus could remove ^137^Cs with above 96% efficiency for less than a 3.16 ppb CsCl loading and the removal efficiency is higher than that reported for two commercialized pot-type water purifiers, composed of activated charcoal and an ion exchanger, or activated charcoal, ceramics and a hollow fiber membrane, with 84.2–91.5% efficiencies for rain water samples [27]. Another set of experiments using commercialized four pot-type purifiers made of materials similar to those above assessed iodine and cesium removability, with efficiencies of approximately 85% and 75–90%, respectively [38]. Others also tested Cs removability using a spongiform adsorbent made of Prussian blue caged within the diatomite cavities and carbon nanotubes, by contacting for 10 hours with low levels of ^137^Cs, yielding a 99.93% removal efficiency [18]. The present test apparatus showed a removal efficiency of over 96% for Cs and I, which is competitive with or better than previously reported removal efficiencies ranging from 75% to 99.93%. It is emphasized here that the advantages of the test apparatus are that it has long been used for domestic use, is easy to operate, provides a sufficient amount of purified water instantaneously (max. 5 l/min.) and offers an established system for proper disposal and/or recycling of used cartridges. Following the FDNPP accident, tap water contamination monitoring revealed that the maximum of the sum of ^134^Cs and ^137^Cs was 180.5 Bq/kg on March 2011 in Tamura, Fukushima Prefecture. It was also reported that a sum of ^134^Cs and ^137^Cs less than 32 Bq/kg was sporadically detected in tap water during 22 days of monitoring after the accident [29]. The water purification plants take precautions not to distribute contaminated water through constant monitoring to meet the latest upper limit value, set by the government as 10 Bq/kg for drinking water, effective from April 1, 2012 [56]. In reality, the detection of greater than 10 Bq/kg radioactivities in tap water in general public is most likely to be the result of accidental and sporadic contamination events. In any case, the test apparatus was demonstrated to decontaminate radiocesium levels to below the detection limit, even when tap water was contaminated by up to 300 Bq/kg radiocesium. When loading 300 Bq/kg of ^137^Cs to the cartridge, the removability obtained was a conditional value of 99.2% and leaving the remaining radioactivity to be below the upper limit value of 10 Bq/kg set by the Government. This indicates that the filtered water right before entering into the electrolysis unit still contains a trace amount of ^137^Cs and the following electrolysis step may produce ^137^Cs enriched ERW. The test apparatus is a powerful electrolysis device yet finely tuned to produce various levels of dissolved hydrogen electric current dependently (Table 1). Under these conditions, we could not definitely exclude a slight possibility that the electrolysis step contributes to ^137^Cs enrichment in ERW. Thus, the only way to clarify such uncertainty was to conduct the experiments as shown in Fig. 5. Moreover, we judged that it is not sufficient enough by just showing the removability of the cartridge filter unit alone and extrapolating the results for evaluating the entire flow-type system. To this end, we decided to measure the radioactivity in ERW, which allows evaluating cartridge unit and electrolysis unit simultaneously. Therefore, the evaluation of the filtering unit in combination with the electrolysis unit as the complete flow-type system was necessary. Our concern for negatively charged I ion was less intense compared to Cs^+^ ion due to higher removability by the cartridge unit and attracted to the anode side. Nevertheless, we confirmed the removability of the flow-type system experimentally to provide the data set with Cs^+^ data.

It is commonly regarded that tap water prepared from lakes and rivers contains varying amounts of organic and inorganic materials. In the present study, we considered these to have a significant impact on removability by the test system because such materials are most likely to compete with the very small amounts of ^137^Cs and ^125^I ions present. Only experiments using low levels of radionuclides will answer the question of whether such interactions between the constituents and added radionuclides may affect removability by this apparatus. Another reason to use lower levels of radionuclides is that even a small amount of ^137^Cs dissolved in water is difficult to remove [11], [20] and accumulates in the body, causing prolonged exposure. Moreover, the fact is that low levels of radioactive Cs species currently contaminate drinking water in many cities around FDNPP [36]. This may be partly attributed to the limited removability of solubilized cesium by the conventional coagulation-sedimentation process [11], [29]. It is therefore extremely important, for the residents of affected regions, to find a way to remove even small amounts of nuclear contaminants from drinking water.

Another concern related to radiocesium is its longer half-life and a characteristic of ready transfer to the human diet through plants [62]. Precipitated Cs^+^ binds to clay minerals rather tightly [27], and depth distribution studies reveal that approximately 80% of total radiocesium is retained in the upper 2.0 cm of tested soil samples [63]. Another study estimated that ^137^Cs could reach a depth of only 18 cm after 300 yr [37]. These characteristics of surface area retention of radiocesium in addition to its long physical half-life (^134^Cs, T_1/2_ = 2.06 yr; ^137^Cs, T_1/2_ = 30.17 yr) could be a secondary contamination source for vegetation via roots. Uptake of radiocesium from the root is thought to occur via the potassium transport system and is distributed rapidly within the plants [62]. Indeed, many agricultural products are reported to be contaminated by radiocesium and are their marketing is restricted [64], [65]. Ingestion of radiocesium-contaminated foodstuffs will expose the gastrointestinal tract and be absorbed into tissues and organs in the body. Gastrointestinal, reproductive and hematopoietic systems are sensitive to ionizing radiation due to their high turnover rate [57], [66]. As an example, the degeneration of small intestinal mucosa cells is caused by free radicals produced from the interactions of radiation energy with intracellular water molecules [66]. Water radiolysis generates a variety of ROS that cause extensive oxidative damages to biologically critical macromolecules, leading to cell death [40], [42]–[45]. Therefore, providing a method to counter radiation hazards caused by accidentally ingested radioactive waters and foodstuffs will be a great contribution to human health.

ERW is regarded as beneficial to health because of its ROS scavenging ability [39]. ERW produced from tap water by this apparatus could contain as much as 0.587 ppm of dissolved hydrogen (Table 1, [48]). This hydrogen concentration in ERW is relatively high for a flow-type electrolysis apparatus when compared with the concentration of 1.6 ppm hydrogen in 100% hydrogen-saturated water [53]. Such dissolved molecular hydrogen has been shown to exert radioprotective effects in both *in vitro* and *in vivo* studies [49]–[53]. Molecular hydrogen in ERW prepared from tap water suppressed neuroinflammation in mice [48], and extended the life span of *C. elegans* [54]. Additionally, molecular hydrogen was demonstrated to act as a neuroprotective agent and ROS scavenger [67]. Moreover, ERW produced from an electrolysis unit incorporating Pt-electrodes has been shown to contain 0.1–0.25 ppb Pt nanoparticles [39], [54], [68]. Pt nanoparticles exhibit protective effects that are attributed to their suppressing ROS production caused by UV-light-induced epidermal inflammation [69]. Synthetic Pt nanoparticles have been shown to scavenge ROS in cultured HeLa cells [70], to induce expression of antioxidant enzyme genes in rat skeletal muscle L6 cells [71], and to act as an SOD/catalase mimetic agent in human lymphoma cells [72]. Model ERW prepared from NaCl, KCl or NaOH solutions has been shown to exert beneficial effects such as anti-diabetic, anti-cancer, and life-span extension of nematodes because of its ROS scavenging ability in numerous *in vitro* and *in vivo* studies [73]–[78]. Therefore, molecular hydrogen and Pt nanoparticles dissolved in ERW could synergistically contribute to protect gastrointestinal damage caused by ingested radioactive foodstuffs. Furthermore, to maximize protective efficacy against radiation-induced gastrointestinal damage, the consumption of various supplemental foods such as naringin [42], probiotics [57], [66], Kefir [79], melatonin [80] and curcumin [81] are reported to be beneficial.

In conclusion, we demonstrated that radio-cesium and -iodine are efficiently removed by an apparatus containing a micro-carbon CM cartridge filter, prior to ingestion. We also suggest that the ERW produced by the test apparatus will provide maximum protection against accidentally and/or unconsciously ingested radionuclides because it contains dissolved hydrogen and Pt nanoparticles. Therefore, the test apparatus is considered to be a potential alternative tool to minimize radiation hazards caused by contaminated foodstuffs.
